# Supplementary material for: Factors associated with pentavalent vaccine coverage among 12-23-month-old children in Afghanistan: A cross-sectional study
Source: PLoS One. 2023 Aug 8;18(8):e0289744. doi: 10.1371/journal.pone.0289744 (PMC10409276; doi:10.1371/journal.pone.0289744)
Supplement: Attachment — Submitted filename: Response to reviewers.docx [file pone.0289744.s001.docx]

**Response to editor’s and reviewers’ comments**

We would like to thank the editor and the reviewers for reviewing our manuscript. We have revised the manuscript according to your comments, which were very helpful. The revised manuscript has been proofread by a native English speaker. The revisions have been completed and the responses are as follows.

**Journal Requirements:**

**1. Please ensure that your manuscript meets PLOS ONE's style requirements, including those for file naming. The PLOS ONE style templates can be found at**

[**https://journals.plos.org/plosone/s/file?id=wjVg/PLOSOne_formatting_sample_main_body.pdf**](https://journals.plos.org/plosone/s/file?id=wjVg/PLOSOne_formatting_sample_main_body.pdf) **and**

[**https://journals.plos.org/plosone/s/file?id=ba62/PLOSOne_formatting_sample_title_authors_affiliations.pdf**](https://journals.plos.org/plosone/s/file?id=ba62/PLOSOne_formatting_sample_title_authors_affiliations.pdf)

We have carefully revised our manuscript to meet PLOS ONE’s style requirements including file names.

**2. You indicated that you had ethical approval for your study. In your Methods section, please ensure you have also stated whether you obtained consent from parents or guardians of the minors included in the study or whether the research ethics committee or IRB specifically waived the need for their consent. Furthermore, you have specified that verbal consent was obtained. Please provide additional details regarding how this consent was documented and witnessed, and state whether this was approved by the IRB.**

Lines 206-212: In the AHS 2018, a verbal informed consent form was used during the data collection. The interviewers read all contents of the form to each participant. When they obtained verbal consent from each participant, they wrote their name and the date of interview, checked the box of “consented”, and signed on the form. When the interviewers collected information about children, they obtained verbal consent from the children’s mother/primary caregiver. We have revised the paragraph of “Ethical issues” as follows, “Verbal consent was obtained from each participant, namely a household head and a mother/primary caregiver of each child, after interviewers read all contents of a verbal consent form to the participant. For data collection of a child, verbal consent was obtained from the mother/primary caregiver of the child. When verbal consent was obtained, the interviewers checked the box of “consented”, wrote their name and the date of interview, and signed on the consent form.”

**3. In your Data Availability statement, you have not specified where the minimal data set underlying the results described in your manuscript can be found. PLOS defines a study's minimal data set as the underlying data used to reach the conclusions drawn in the manuscript and any additional data required to replicate the reported study findings in their entirety. All PLOS journals require that the minimal data set be made fully available. For more information about our data policy, please see** [**http://journals.plos.org/plosone/s/data-availability**](http://journals.plos.org/plosone/s/data-availability)**.**

The data for AHS 2018 is a property of the Ministry of Public Health (MoPH) of Afghanistan. MoPH of Afghanistan has the ownership and authority to manage the AHS 2018 data. Only authorized and permitted researchers by MoPH are allowed to access and use the data for research and analytical purposes. Data are available from the Ministry of Public Health of Afghanistan (General Directorate of Monitoring & Evaluation and Health Information System; Tel +93-20-231-3688 | Emails [ehis.moph@gmail.com](mailto:ehis.moph@gmail.com), mehis.moph@gmail.com) for researchers who meet the criteria for access to confidential data.

**Reviewer #1:**

**Introduction**

1. **What is the difference between administration and coverage? Please provide the definition of vaccine coverage in your introduction.**

We used the term “administration” as the process of applying vaccine for the targeted children and the term “coverage” as the outcome of applying vaccine for the target children. We think that “coverage” is more appropriate rather than “administration” in our manuscript and we have revised “administration” to “coverage” in the manuscript.

2. **Just to reinforce, it’s a suggestion: Please provide information about the EPI centers, their establishment and operations, if vaccine administration is provided free of charge by the state.**

Line 80-83: Immunization is one of the key components of Basic Package of Health Services in Afghanistan and provided at EPI centers free of charge. EPI centers have at least one trained vaccinator to apply the routine immunization vaccines to under-five children on a daily basis. The number of EPI centers increased from 1,575 in 2015 to 2,926 in 2018 and EPI centers cover all areas in the country. We have added as follows, “Since the EPI started in Afghanistan, the vaccine delivery system has been improving. EPI centers, which have at least one trained vaccinator, provide the routine immunizations free of charge and the number of EPI centers increased from 870 in 2004 to more than 1,767 in 2016 [13].”

3. **UNICEF/WHO target of immunization is not mentioned in the manuscript, add it please.**

Lines 76-78: The WHO Eastern Mediterranean Region (EMRO) set the 90% vaccination target for member countries. We have added a sentence as follows, “The WHO Eastern Mediterranean Region set the target of childhood vaccine coverage for all antigens as 90% for countries in the region, including Afghanistan [11].”

4. **L65-66: suggestion change (ages 1-4) by (under five years).**

Line 65-66: We have revised “child mortality rate (CMR) (ages 1-4)” to “mortality rate of children under five years old (under-five mortality).”

5. **L67: please use updated information if available, 2015 to today many things happened.**

Line 66-68: The World Bank estimates the mortality rate of children under five years old in Afghanistan and the latest estimation reported 58 per 1,000 live births in 2020. We have revised the sentence as follows, “the under-five mortality in Afghanistan declined from 191 per 1,000 live births in 2006 to 58 per 1,000 live births in 2020 [8,9].”

6. **L78-80: Please provide the national vaccination coverage estimate. If possible, show how it improved from 2015 to 2018 and wish regions of Afghanistan present the lowest coverage.**

Line 80: “Immunization services” means “vaccine delivery system” and we have revised as follows, “the vaccine delivery system has been improving.”

Lines 83-85: According to the results of the nation-wide surveillance, the routine immunization coverage increased from 30.1% in 2003 to 59.7% in 2013. However, the coverage was 58.6% in 2015, which decreased to 50.2% in 2018. The coverage was especially low in southern and southeastern provinces (Helmand, Zabul, Urozgan, and Paktika Provinces) (3.1%-28.1%). We have added a sentence as follows, “The routine immunization coverage improved from 30.1% in 2003 to 59.7% in 2013 [10], but decreased from 58.8% in 2015 and 50.2% in 2018 [8, 14]. The coverage in 2018 was low (3.1-28.1%) in southern and southeastern provinces.”

7. **L80-83: suggestion:** **The factors associated with non- and under-vaccination of children in Afghanistan were reported to be socio-demographic and childhood factors, lack of urbanization, access to health facilities, and household factors including maternal and paternal education.**

Line 92-95: According to the reviewer’s comment, we have revised as follows, “the factors associated with non- and under-vaccination of children in Afghanistan were socio-demographic and childhood factors, lack of urbanization, access to health facilities, and household factors including maternal and paternal education.”

8. **L83-85: The justification can be improved, data regarding vaccination coverage using community data are available in Afghanistan, it would be important to provide a strong justification and show which gap your study will fill.**

Line 80-98: The vaccine service system has been developing, but Afghanistan has still outbreaks of infectious diseases which can be prevented by the 3 doses of pentavalent vaccine (Penta3). Vaccine service by mobile health teams was introduced in 2003 but the coverage of Penta3 was not higher in the areas with mobile health team services compared to the areas without the services. Therefore, factors influencing lower coverage of Penta 3 should be understood but no studies have been published focusing on factors associated with Penta3 coverage among children in Afghanistan. We have revised the part of the paragraph as follows, “Since the EPI started in Afghanistan, the vaccine delivery system has been improving. EPI centers, which have at least one trained vaccinator, provide the routine immunizations free of charge and the number of EPI centers increased from 870 in 2004 to more than 1,767 in 2016 [13]. The routine immunization coverage improved from 30.1% in 2003 to 59.7% in 2013 [10], but decreased from 58.8% in 2015 and 50.2% in 2018 [8, 14]. The coverage in 2018 was low (3.1-28.1%) in southern and southeastern provinces. Therefore, Afghanistan still has strong outbreaks of vaccine-preventable diseases, especially diseases that can be prevented by Penta. In 2003, providing maternal and child health services by mobile health teams, which consist of a midwife, a vaccinator, and a nurse, started in remote and conflict-affected areas. In 2016 and 2017, the coverage of Penta3 in the areas with mobile health team services was 26.6%, which was lower than that in the areas without the services (30.5%) [15]. To improve the coverage of Penta3, the factors affecting no or incomplete vaccination of Penta3 should be understood. Previous studies reported that the factors associated with non- and under-vaccination of children in Afghanistan were socio-demographic and childhood factors, lack of urbanization, access to health facilities, and household factors including maternal and paternal education [2,5,12]. However, to our knowledge, there have been no studies on the factors associated with Penta3 coverage in Afghanistan. Therefore, this study aimed to identify factors associated with Penta3 coverage among children aged 12-23 months in the country, using the data of the Afghanistan Health Survey (AHS) 2018.”

9. **L84: you have Penta3 administration, however, in L86 coverage, please standardize.**

Line 96: As we have stated to a previous comment above, we have revised “administration” to “coverage” throughout the manuscript.

**Materials and Methods:**

10. **For better visualization please provide a map that locates it on the continent, as well as the division by provinces.**

Line 104: Thank you very much for your comment. We use “Afghanistan Health Survey 2018” as a reference (No. 14), which explains the sampling methods and includes maps of provinces of Afghanistan. Therefore, everyone can see the maps by searching “Afghanistan Health Survey 2018” without a figure of a country map in the manuscript. For this reason, we do not feel that it is necessary to include a figure of a map.

11. **The time of data collection is not mentioned by the authors the survey took a years or a couple of months? How did they collect it? by visiting individual home? What was the response rate of the participating mothers? method section lacks this information.**

Lines 109-110: Data collection took place between March 2018 and August 2018. Field teams consisted of male and female interviewers who visited each selected household and interviewed all men who have ever been and women in the household after obtaining verbal consent. The report of AHS showed the response rate of households and women and the complete rate of children’s data (anthropometric measurement and health data). Furthermore, children included in the AHS 2018 were under five years old but our study included children aged 12–23 months old. Therefore, we could not find the response rate of mothers/primary care givers of children 12-23 months old. We have revised the method section as follows, “Data collection was conducted from March to August in 2018 [14]. Field teams consisted of male and female interviewers, recruited from each of the 34 provinces of Afghanistan. All staff received extensive training prior to going into the field. The AHS instruments consisted of six questionnaires: (1) general information and socio-demography, (2) household history, (3) anthropometry of children, (4) mother’s general information, (5) women’s pregnancy history, and (6) child immunization status. The field teams visited each selected household and interviewed all men who had ever been married and women aged 15-49 of the household using the questionnaires after obtaining verbal consent. The response rate of households and women was 94.4% and 93.9%, respectively [14]. Regarding children’s data collection, the anthropometric completion rate and child health completion rate was 93.8% and 94.3%, respectively. Data collection in the field took place by adequate interview techniques maintaining all ethical protocols. When the data were analyzed, sampling weights were applied in order to adjust the results for differences that might be caused by the selection steps.”

12. **L91-92: Change by Cross-sectional study, conducted by the MoPH……………**

Line 102: The manuscript has been proofread by a native English speaker. According to the English editor’s advice, it would be better to keep the original sentence.

13. **L98-99: please make it clear**

Line 116-120: We understand that the reviewer asked about “for security reasons and the safety of data collectors.” The security issues were recorded active fights (civil wars) in the selected clusters; therefore, female team members were not allowed to enter the area. The survey team was not allowed to use electronic devices. Furthermore, access to the area was difficult because of the geographical terrain. We have revised the sentence as follows, “However, less than two-thirds of the selected clusters were surveyed in Zabul, Nuristan, and Helmand Provinces due to civil wars and hard-to-reach areas [14].”

14. **L116-L118: Suggestion: The characteristics of the 12–23-month-old children, their households, characteristics of their household-heads and mothers/caregivers were taken from the AHS database.**

Lines 138-140: The manuscript has been proofread by a native English speaker. According to the English editor’s advice, it would be better to keep the original sentence.

15. **L124-125: In Afghanistan, the Vitamin A administration is made only in National campaigns and not as a routine immunization in Health Center? And this information usually goes to child health cards, why this information was only obtained through interviews. Please check L296**

Lines 149-150: In Afghanistan, vitamin A is provided to children by the National Immunization Campaigns but not the routine immunization. The child vaccination card for routine immunization in Afghanistan does not record the administration of vitamin A. Therefore, data collectors asked mothers/primary caregivers if their child took vitamin A or not. We have added as follows, “In Afghanistan, vaccine record cards do not record vitamin A supplementation.”

16. **L133: please check if is “number of total eligible women” or “number of total eligible children”.**

Line 156: We have checked and “number of total eligible women” is correct.

17. **L153-162 and 163-170 check if the information is not overlapped, what is the difference between these two paragraphs and why they are important?**

Lines 176-185 and 186-193: The first paragraph (lines 176-185) explains about variables for the data of the heads of household and the second paragraph (lines 186-193) explains about variables of the data mothers/primary caregivers. Age and the educational history were included in both data but the methods of categorization were different. Variables other than age and the educational level were different between household heads and mothers/primary caregivers. Therefore, these two paragraphs are needed and important to explain about variables and categories.

18. **L173 there is no information about the descriptive analysis, please add.**

Lines 196-197: We have added the sentence as follows, “Descriptive analysis was used to describe the characteristics of children, their households, and their household heads and mothers/caregivers.”

**Results:**

19. **L187: something is missing after the comma**

Line 215: We have checked the sentence carefully and there is nothing missing after the comma.

20. **190: please remove the word “old”**

Lines 217-218: “1,742 children (57.3%) were 12–17 months old” and “the average age was 16.5 months old” are grammatically correct.

21. **191: Please check the percentage of children without vaccination card, because in line 191 you have 11.2%, while in the limitation section 12.0%, please revise.**

Line 387: We are sorry that the percentage in the limitation section was incorrect. We have revised it from 12.0% to 11.2%.

22. **General observation: how did you conduct the descriptive analysis on the tables? I was expected to see each one of the values divided by the sample size, not by its own “n”. For example, in table 1, I was expected to see diarrhea variable 68.4% for “no” and “31.6%” for yes, which reflects the estimates that you have in the country survey. This is just an observation the authors can think and discuss about it.**

Tables 1-3: The percentage of “Penta3” shows the proportion of children who had Penta3 among total children in each category. We have added the number and percentage of children who did not have Penta3 (who had 0-2 doses of pentavalent vaccine).

23. **191-196: I think it deserves a subtitle maybe “Vaccination coverage” and concentrate all the information regarding the vaccine coverage.**

This is a part of the result section and subtitles are not used in the result section. The percentage of columns of “Penta3” means the coverage of Penta3 among each category in Tables 1-3. The comparison of the coverage among categories of each variable is explained from page 9 to page 12.

**Discussion**

24. **Make a comparative assessment of vaccination coverage in Afghanistan and other countries in the region.**

Lines 360-369: In our study, the Penta3 coverage among children 12-23 months old. It is difficult to find the same coverage in other countries. Therefore, we compared the coverage of DPT/Penta3 coverage among 21 member countries in the WHO Eastern Mediterranean region using the data by the WHO. We have added a paragraph in the discussion section as follows, “In the WHO Eastern Mediterranean region including 21 member countries, not only Afghanistan but also six other countries did not achieve the target of the DPT/Penta3 coverage among children under one year old in 2018; 87% in Afghanistan, 84% in Djibouti and Iraq, 80% in Yemen, 72% in Pakistan, 69% in Somalia, and 66% in Syrian Arab Republic [37]. These results suggest that living in conflict-affected areas may be a strong risk factor for low DPT/Penta3 coverage in the region by causing cancellation of planned services and missed opportunities. Humanitarian crisis, such as wars and natural disasters, are one of major barriers for improving the vaccine coverage [38]. Identifying children in hard-to-reach areas and expanding vaccine services to children who are older than the target age should be focused on more to improve the coverage.”
