# Supplementary material for: Factors associated with pentavalent vaccine coverage among 12-23-month-old children in Afghanistan: A cross-sectional study
Source: PLoS One. 2023 Aug 8;18(8):e0289744. doi: 10.1371/journal.pone.0289744 (PMC10409276; doi:10.1371/journal.pone.0289744)
Supplement: Attachment — Submitted filename: Response to Reviewers.docx [file pone.0289744.s002.docx]

**Response to editor’s and reviewers’ comments**

We would like to thank the editor and the reviewers for reviewing our manuscript. We have revised the manuscript according to your comments, which were very helpful. The revised manuscript has been proofread by a native English speaker. The revisions have been completed and the responses are as follows.

**Journal Requirements:**

**1. Please review your reference list to ensure that it is complete and correct. If you have cited papers that have been retracted, please include the rationale for doing so in the manuscript text, or remove these references and replace them with relevant current references. Any changes to the reference list should be mentioned in the rebuttal letter that accompanies your revised manuscript. If you need to cite a retracted article, indicate the article’s retracted status in the References list and also include a citation and full reference for the retraction notice.**

We are very sorry that we did not explain about changing references or adding references when we submitted revised manuscript. It is because we used EndNote (a software for references). When EndNote is used for references, revisions of references and reference numbers cannot be found even if the function of “track change” is used in WORD file. Therefore, we have added underlines for the revised reference numbers in the manuscript and for the revised reference (No. 9) in the reference section.

**Additional Editor Comments:**

2. **Please make subdivisions on the abstract as one reviewer points out.**

We have added subheadings in the abstract.

3. **Line 68 there is one citation pointing to the World Bank as the source of child mortality estimates. This is incorrect. Please a UN IGME reference for this.**

Line 68 and reference No. 9: We have revised the reference (No. 9) for under-five mortality rate in Afghanistan from the World Bank to United Nations Inter-agency Group for Child Mortality Estimation.

**Methods:**

4. **About the AHS 2018 sampling. With the current description, it seems that there is province stratification. How about urban/rural?**

Lines 106, 109-110: We have added the explanation of stratification considering urban and rural areas and the number of households in urban and rural area as follows, “Stratification was achieved by separating each province into urban and rural areas. “ (line 106) and “Therefore, 23,460 households (4,738 households in urban areas and 18,722 households in rural areas) were selected as the survey samples in all 34 provinces.” (lines 109-110).

5. **Line 113/114: the last sentence is a repetition of a sentence in 110/112. Please, revise.**

We have found that two sentences were repeated in the paragraph and we have erased them.

6. **There were 3 provinces that did not reach the desired sample size. What was done for these provinces at the analysis level?**

We applied sampling weights when we performed analyses, because sampling weights were calculated according to two types of probability of selection, including selection by design and by factors other than design (affected by non-response of a respondent). Taking into account response rates in the weights, makes it also inevitable to introduce individual weights, in addition to household weights. The fact that the questionnaires were designed to interview different types of respondents for household, eligible women and children under 5, anthropometric, and verbal autopsy sections, requires separate response rates for these types of respondents. In AHS, these response rates are calculated at the stratum level. The combination of these two factors, design and non-response, produces household and different individual weights.

7. **What languages were used to talk to the mothers? Were the data collectors trained for this?**

Lines116, 186-188: The questionnaire forms were translated to Dari and Pashto. When literacy of mothers/primary caregivers was estimated, the data collectors observed their reading and writing ability of any of the common languages of the country (Pashto, Dari, or Uzbek language). Extensive training was provided to trainers and field staff prior to going into the field. The detail methods of trainings of data collectors are explained in the report of AHS 2018, which is included as a reference in our manuscript (No. 14).

8. **In a household with more than 1 child eligible to enter the sample what was done? This is an important detail to describe.**

Lines 131-133: All households included in the AHS 2018 had only one eligible child (12-23-month old). We explained as follows, “Of the 19,684 households included in the AHS 2018, 3,040 households had a child aged 12–23 months and there was no household that had two or more children aged 12–23 months.”

9. **Did the analysis use survey weights or not? This seems to be ignored in the whole analysis.**

Yes, we used sampling weights in the analyses.

10. **Line 180: The categorization of age obeys quantiles of age. What this means quartiles, quintiles? The below age 29 may include families led by someone under-18 or under-21. This may matter at least for description.**

We divided age of household heads into the four groups and the interval was 15 years. Each category suggests characteristics of people. “≤29 years” means the young generation, “30–44 years” means the generation of reproductive, active, and having experiences, “45–59 years” means “senior but still active”, and “≥60” means “after retirement and grandparents”. It is not so rare in Afghanistan that household heads are under18 years or 21 years because of conflicts or terrorist attacks. In our study, household heads who were under 18 years and 21 years were 8 (0.3%) and 40 (1.3%), respectively. In lines 248-249, we have added a sentence as follows, “There were 8 (0.3%) and 40 (1.3%) household heads who were under 18 and 21 years, respectively.”

11. **About the "ethical issues". This report is a result of a secondary data analysis of AHS 2018, correct? If yes, clarify that the ethical approval you got was to access the data not to conduct the data collection.**

Lines 138-139, 207-209: This study conducted a secondary analysis of the data of AHS 2018 and we did not do data collection. Our study was approved by IRB of the MoPH in Afghanistan. The first author used to work for the MoPH in Afghanistan. For his PhD study, he requested the AHS dataset to General Directorate of Monitoring & Evaluation and Health Information System, MoPH and it was accepted. In lines 138-139, we have added the following sentence, “The dataset was provided by the MoPH for this study.”

**Results:**

12. **Please do not use only p-values on table 1 through table 3 (it would be fine to remove these p-values). In fact, many of these p-values are repeated in the unadjusted analysis of table 4. For tables 1 to 3 do: 1) make the percentages to be by column; 2) add a column for coverage and its 95% confidence interval.**

Tables 1-3: This style of tables and a combination of chi-square test/Fisher’s exact test and logistic regression analysis are commonly used in many papers that were published not only from PLoSOne but also other journals. The coverage of Penta3 in each category is easy to understand in Tables 1-3, because it is written as the percentage of children in the column of Penta 3 “Yes.”

13. **Table 4 - there is nowhere in the manuscript explaining how the factors were chosen. Is this based on the literature? Please state so. Why not adjust by province?**

Lines 192-193: The variables were chosen according to previous studies on vaccination coverage. We have added the following sentence, “Variables for this study were chosen according to previous studies on the vaccine coverage [2, 5, 12, 18-21].”

There are 34 provinces in Afghanistan. We would like to perform analyses to understand the vaccine coverage and associated factors in the national level but the provincial level. Therefore, province was not included in variables in our study.

**Reviewer #2:**

14. **Title: The title is good but why you prefer only coverage of PCV3 vaccine coverage? Why you ignore PCV1, PCV2….???**

It is because three doses of pentavalent vaccine are recommended and scheduled in the EPI schedule in Afghanistan.

15. **Your source of data is not clear; is it primary data from face to face interview or secondary data from previous records??? Be consistent and revise it throughout the document. If you say it was secondary data how do you get consent from participants?**

Line 102: We have moved the sentence “This study is a secondary analysis of the AHS 2018 data” to the beginning of the method section, to emphasize that this study conducted a secondary analysis.

Lines 207-209: In the AHS, informed consent was obtained from each participant in the AHS. In this study, informed consent was waived due to a secondary analysis of anonymous data of the AHS and the study protocol of this study was approved by IRB. We have revised the sentence as follows, “In this study, written informed consent was waived due to a secondary analysis of the anonymous data of the AHS 2018 and the study protocol was approved by the Institutional Review Board of the MoPH in Afghanistan (Approval number: E.1019.0087).”

16. **The variables like diarrhea, bipedal edema, vitamin A supplement, smoking of heads of households,… are irrelevant and clinically insignificant variables for vaccine coverage so why you include this factors on multivariable regression? Because inclusion of such irrelevant variables in the analysis might confound the effect of other variables.**

In a setting like Afghanistan, children’s nutrition status and immunity level, and parents’ behavior and habits are highly connected with immunization and health status of under five children. These are reported in various studies and reports in Afghanistan and other developing countries. Furthermore, in AHS 2018, the immunization coverage of the children was evaluated and was associated with the vitamin A supplementation, diarrhea history and nutrition status of the children. In various reports and studies, parents’ habits such as smoking were associated with education level, child health statues, immunization, and health practices in the household. Therefore, we believe that it is necessary to include household factors (for example; smoking cigarettes by the head of the household), because the results increase the awareness of improving desirable behavior and habits among the parents and give an alert to the Ministry of Health of the country and legislation authorities. It is easy and cheap to purchase (2 or more boxes of cigarettes cost 1 USD) and smoke cigarettes without considering any public health and community norms. Considering these facts and results, we included diarrhea, bipedal edema, vitamin A supplement, smoking of heads of households in this study.

17. **Variables of the study should better to be summarized under one or two paragraphs and operational definitions should be stated in separate section.**

We used four groups of variables, namely variables of children, variables of households, variables of household heads, and variables of mothers/primary caregivers. We made three paragraphs with titles of variable groups. In each paragraph, variables are listed and definitions or categories are explained. Before starting explanation about variables, we wrote “The characteristics of the 12–23-month-old children, the characteristics of their households, and the characteristics of their household-heads and mothers/caregivers were taken from the AHS dataset.” Therefore, we think that our writing in the method section is also understandable for readers.

18. **How do you check multicollinearity? Better to incorporate it in the manuscript.**

Lines 199-202: We have checked multicollinearity and added the following sentences as follows, “Multivariate logistic regression analysis on Penta3 included 18 variables that showed significant differences by a Chi-square test. Multicollinearity was not found between the variables according to the variance inflation factor values for each of variables.”

19. **Almost all tables are mere repetitions of text descriptions, better to avoid redundancy by choosing either of the two.**

We think that the result section is a part to explain results of analysis (tables), therefore, we explained the results in tables in the section.

20. **Variables such as; diarrhea, bipedal edema, vitamin A supplement and smoking of heads of households are not strongly justified for its association with vaccine coverage in the discussion section. revise it again.**

No diarrhea in the last 2 weeks (P=0.005), presence of bipedal edema (P=0.019), vitamin A supplement (P<0.001), and no smoking of heads of households (P=0.017) were significantly associated with having Penta3, because the P-value was less than 0.05. Therefore, we included these variables in the discussion section.

**Reviewer #3:**

21. **The abstract section lacks sub heading. The abstract section is lengthy.**

We have included subheadings in the abstract.

22. **The introduction section is also too long. It should address the tried effort to decrease the burden of the problem your country, current intervention, and any future directions. Furthermore, you should give a strong gap. Your gap is not convincing.**

We revised the introduction section in order to response to the comments from Reviewer 1, therefore, the section may be long. We would like reviewers and the editor to understand how we revised according to the comments from Reviewer 1.

23. **In the method section, you merged method sub-sections. You need to separate and present in clear way. Data collection method, sampling procedure, Tool.**

We explained the AHS 2018 in the first paragraph of the method section. Therefore, data collection method, sampling procedure, and the tool of the AHS 2018 were explained in this paragraph. After the first paragraph in the method section, we explained study participants and variables of our study separately in each paragraph.

24. **There are a lot of repetitive ideas. E.g. the study period is presented in page 3 line number 103, 115....**

We have erased some sentences to avoid repetition.

25. **Is your tool valid and reliable? Is it developed by the authors or adopted?**

This study is a secondary analysis of the AHS data and the tool was developed and validated in the AHS but not us.

26. **Check the whole document for typo errors. Be consistent with use of grammars. Statistical analyses vs statistical analysis?**

We have carefully checked typo errors and revised from “analyses” to “analysis” in the three parts.

27. **In the result section: you need to avoid repetition of data already stated in the table. Make it short and precise.**

The result section is the part to explain the results of analysis (tables), therefore, we explained the results in tables in the section.

28. **Strong recommendation: The result section should be written in short and precise way.**

We have revised the result section and tried to make shorter.

29. **Have you checked the model assumptions? if so report the findings.**

Lines 199-202: We have checked multicollinearity and added the following sentences as follows, “Multivariate logistic regression analysis on Penta3 included 18 variables that showed significant differences by a Chi-square test. Multicollinearity was not found between the variables according to the variance inflation factor values for each of variables.”

30. **In the discussion section: please check the typing errors. You need to discuss the implication of the finding.**

We have checked typo errors throughout the manuscript. We discussed the implication of the findings (Pent 3 coverage and factors associate with Penta 3) in the discussion section.

31. **The declaration section seems incomplete.**

We have revised “Ethics Statement” in the submission process. We will follow the guidance by the journal office if any revision is required.
