# Supplementary material for: Factors associated with pentavalent vaccine coverage among 12-23-month-old children in Afghanistan: A cross-sectional study
Source: PLoS One. 2023 Aug 8;18(8):e0289744. doi: 10.1371/journal.pone.0289744 (PMC10409276; doi:10.1371/journal.pone.0289744)
Supplement: Attachment — Submitted filename: Response to Reviewers.docx [file pone.0289744.s003.docx]

**Response to editor’s comments**

We would like to thank the editor and the reviewers for reviewing our manuscript. We have revised the manuscript according to your comments, which were very helpful. The revisions have been completed and the responses are as follows.

**1. Please do not use only p-values on table 1 through table 3 (it would be fine to remove these p-values). In fact, many of these p-values are repeated in the unadjusted analysis of table 4. For tables 1 to 3 do: 1) make the percentages to be by column; 2) add a column for coverage and its 95% confidence interval. The authors state that it is a matter of style. This is a serious misconception. This is a succinct presentation of the current results in this manuscript. The reader would benefit if the row percentages was presented as coverage [the column I am suggesting to add]; and the characteristics were presented in column percentages. The p-values have no role here.**

According to the editor’s comment, we have revised Tables 1-3 by erasing p-values by chi-square test and adding the number and percentage (and 95%CI) of children who completed Penta3.

**2. Also, pay attention to reviewer 3 in the last revision comments (as per his/hers recommendation). Particularly, please, respond fully to the following questions: The question about model assumption was not fully responded to. At least please state how did you check model fit which is closer to model assumptions check requested by the reviewer. Yes, checking multicollinearity is important but it is not enough.**

We have checked the model assumption by Hosmer-Lemeshow test and it showed that the force-entry method was not a good fit. Therefore, we performed multivariable stepwise logistic regression analysis with forward-selection and backward-selection and Hosmer-Lemeshow test showed that these two models were a good fit. In Table 4, we have erased the results of the force entry method and added the results of stepwise forward-selection and backward-selection methos. We have also added the results of Hosmer-Lemeshow test in the footnote of Table 4. We have revised the abstract, method, result, and discussion sections according to the change of multivariable analysis.

**3. Change the term multivariate to multivariable.**

We have revised “multivariate” to “multivariable” in the manuscript.
